# Under the Shadow: Old-biased Genes Are Subject to Weak Purifying Selection at Both the Tissue- and Cell Type-Specific Levels

**DOI:** 10.1093/gbe/evaf187

**Published:** 2025-10-15

**Authors:** Nisan Yıldız, Hamit İzgi, Firuza Rahimova, Umut Berkay Altıntaş, Zeliha Gözde Şahin, Mehmet Somel

**Affiliations:** Department of Biological Sciences, Middle East Technical University, Ankara 06800, Turkey; Department of Biological Sciences, Middle East Technical University, Ankara 06800, Turkey; Department of Biological Sciences, Middle East Technical University, Ankara 06800, Turkey; Department of Biological Sciences, Middle East Technical University, Ankara 06800, Turkey; Department of Biological Sciences, Middle East Technical University, Ankara 06800, Turkey; Department of Biological Sciences, Middle East Technical University, Ankara 06800, Turkey; Swedish Collegium for Advanced Study, Uppsala, Sweden; Department of Archaeology and Classical Studies, Stockholm University, Stockholm, Sweden

**Keywords:** evolution of ageing, transcriptomics, mutation accumulation, selection shadow, single-cell, cell type composition

## Abstract

The mutation accumulation hypothesis suggests that weakened purifying selection at old age leads to the accumulation of late-acting deleterious variants in the gene pool, which may contribute to the evolution of ageing. In accordance with this model, others and we have shown that sequence conservation among old-biased genes (with higher expression in old vs. young adults) is weaker than among young-biased genes across several mammalian and insect species. However, it has remained unclear whether the observed patterns were driven by tissue and cell type composition shifts or by cell-autonomous expression changes during ageing. How wide this trend would extend to nonmammalian metazoan ageing was also uncertain. Here we analyzed bulk tissue as well as cell type-specific RNA sequencing data from diverse animal taxa across six different datasets from five species. We show that the previously reported age-related decrease in transcriptome conservation is commonly found in ageing tissues of nonmammalian species, including nonmammalian vertebrates (chicken brain, killifish liver and skin) and invertebrates (fruit fly brain). Analyzing cell type-specific transcriptomes of adult mice, we further detect the same age-related decrease in transcriptome conservation trend at the single-cell type level. Old-biased genes are less conserved across most cell types analyzed in the lung, brain, liver, muscle, kidney, and skin, and these include both tissue-specific cell types, and also ubiquitous immune cell types. Our results support the notion that ageing in metazoan tissues is shaped by the mutation accumulation process.

Significance Extensive theoretical work and experimental studies on model organisms have shown that purifying selection cannot effectively remove harmful variants when they are expressed at old age (i.e. ages when most individuals would be expected to die due to extrinsic causes, such as predation). This phenomenon is called the “selection shadow”. Furthermore, previous work had shown lower conservation (weaker purifying selection) on genes expressed among old adults than in young adults, but this was conducted using whole tissue transcriptomes composed of multiple cell types, and had been only performed in mammals and insects. Here we use cell type-specific transcriptomes from six different mouse tissues to show that signs of the selection shadow can be observed directly within specific cell types. We also report selection shadow signatures in a diverse group of metazoan bulk-tissue transcriptomes, including in chicken, turquoise killifish, and fruit flies. Our results highlight the generality of the selection shadow effect in metazoan ageing.

## Introduction

Ageing is defined as progressive decline in fitness with increasing age (Kirkwood and Austad 2000; López-Otín et al. 2013). The mutation accumulation theory provides a simple and powerful explanation of how ageing could evolve (Medawar 1952). This is based on the premise that in many natural populations, the number of individuals contributing to the next generation declines with age by extrinsic mortality, even without invoking the intrinsic effects of ageing. Consequently, phenotypically deleterious variants that show their deleterious effects only at old ages, when the residual effective population size is low, can drift to fixation, creating the so-called “selection shadow” at late ages [reviewed in (Flatt and Partridge 2018)]. The accumulation of such late-acting deleterious variants could then explain the prevalence of ageing phenotypes, leading to Gompertzian mortality curves at old ages. Conversely, ageing could be absent in species in which the probability of extrinsic mortality decreases with age, e.g. in species where individuals can constantly grow in size. This prediction is supported by empirical evidence: in diverse animal species, mortality can be stable or even decline through lifetime (Jones et al. 2014; Cohen 2018).

Testing the role of the mutation accumulation mechanism on ageing initially relied on studying inter-individual heterogeneity of age-related fitness reduction, a prediction of the model (Charlesworth and Hughes 1996; Shaw et al. 1999; Wilson et al. 2007; Escobar et al. 2008). More recently, researchers have also begun employing molecular data to test the idea. For instance, Rodríguez and colleagues used genetic disease and polymorphism data in humans to show that late-acting disease variants segregate at higher frequencies than early-expressed variants (Rodríguez et al. 2017).

Yet another line of studies has combined sequence divergence data (*dN/dS* ratios; ratio of rate of nonsynonymous mutations to synonymous mutations) and transcriptome data, under the assumption that old-biased genes will carry late-acting variants. These studies showed that genes with increased expression levels in old versus young adults (old-biased genes) tend to be less conserved than young-biased genes in human brain ageing (Somel et al. 2010), and across human bulk tissues (Jia et al. 2018). Recently, we systematically investigated the pattern of lower evolutionary conservation of genes expressed at late age using 66 transcriptome datasets representing human, macaque, mouse and rat bulk-tissue samples (Turan et al. 2019). We identified the same phenomenon, which we termed age-related decrease in transcriptome conservation (ADICT), in 76% of these datasets. Interestingly, although some tissues, such as brain, lung and liver, showed conspicuous ADICT signatures, other tissues, such as muscle and heart, revealed no consistent pattern. We further found old-biased and weakly conserved mammalian genes to be enriched in apoptosis and inflammation-related processes.

More recently, Cheng and Kirkpatrick reported low conservation of old-biased genes across human, mouse, fruit fly and mosquito transcriptomes (Cheng and Kirkpatrick 2021), also showing that old-biased genes carry higher levels of functional polymorphism (pN/pS) and tend to be evolutionarily younger. Meanwhile, Harrison and colleagues studied sequence conservation of young- and old-biased genes identified in whole-body transcriptomes in relatively long-lived ant queens (*Cardiocondyla obscurior*) (Harrison et al. 2021). Intriguingly, these authors found higher conservation of old-biased genes, a result which would be consistent with the reported absence of reproductive senescence in these ant queens. In another study, Cui et al. compared protein coding sequences between short- and long-lived killifish and reported higher conservation of genes expressed at young age, as well as genome-wide signatures of relaxation of selection in short-lived killifish (Cui et al. 2019).

This growing body of evidence suggests a role for Medawar's mutation accumulation process, and specifically, a role for higher drift on old-biased genes in metazoan ageing. Still, caution is warranted. First, the diversity of taxa studied is limited to five species of mammals, one fish species, and three species of insects. Second, studies conducted hitherto have analyzed either bulk tissue (mammals and killifish) or whole-body samples (insects). It therefore remains possible that the observed expression trends, and expression-conservation correlations, may in fact be driven by ageing-related changes in tissue composition and/or cell type composition within tissues. Indeed, ageing causes apparent shifts in cell type composition, including tissue-specific cells and immune cells, as shown in mouse tissues (Tabula Muris Consortium 2020), in the human brain (Soreq et al. 2017) and in the fruit fly brain (Davie et al. 2018). Shifts in a tissue's cell type composition with age will likewise shift expression patterns measured at the bulk-tissue level, even in the absence of cell-autonomous expression change. Furthermore, tissues are known to vary in the average conservation levels of the genes they express (Khaitovich et al. 2005), and we may reasonably expect that cell type transcriptomes likewise vary in their average conservation levels. Accordingly, the observed ADICT pattern can have two, nonmutually exclusive explanations: (i) old-biased genes at the cell-autonomous level being subject to stronger drift and (ii) weakly conserved cell types increasing their representation within tissues at late age.

Here we address these issues, first by studying the prevalence of ADICT across a diverse range of organisms using published bulk-tissue transcriptome profiles (as opposed to whole organism transcriptomes). Second, we investigate conservation level differences among mouse cell types, and we test whether ADICT can be observed at the cell type-specific level. We perform this using two alternative approaches, one that uses expression-conservation-age correlations, and one that divides genes into young- and old-biased classes and compares conservation between the two. We further use conservation estimates based on inter-species divergence or on population genomics data.

## Results

### Age-related Decrease in Transcriptome Conservation is Observed Across Diverse Metazoan Species

We first asked whether the previously described pattern of age-related decrease in evolutionary transcriptome conservation (ADICT) (Turan et al. 2019) can also be observed in tissue-specific transcriptomes of nonmammalian organisms. To this end, we collected published ageing transcriptome datasets from diverse taxa, including a *G. gallus* (chicken) brain ageing dataset, a *N. furzeri* (turquoise killifish) liver and skin ageing dataset, and a *D. melanogaster* (fruit fly) brain ageing dataset (Table 1).

**Table 1:** Summary of gene expression datasets used in this study

Organism	Age groups (sample size per tissue)	Tissue	RNA-seq type	Accession	Reference
*Drosophila melanogaster*	5-days-old (*n* = 6), 20-days-old (*n* = 6), 30-days-old (*n* = 5), 40-days-old (*n* = 6)	Brain^a^	Bulk	DOI: https://doi.org/10.1371/journal.pone.0209405	Pacifico et al., (2018)
*Nothobranchius furzeri*	5-weeks-old (*n* = 5), 12-weeks-old (*n* = 5), 20-weeks-old (*n* = 5), 27-weeks-old (*n* = 5), 39-weeks-old (*n* = 5)	Liver, Skin	Bulk	GSE: https://www.ncbi.nlm.nih.gov/geo/query/acc.cgi?acc=GSE66712	Reichwald et al., (2015)
*Gallus gallus*	100-days-old (*n* = 3), 300-days-old (*n* = 3), 365-days-old (*n* = 3), 1095-days-old (*n* = 2), 1825-days-old (*n* = 2)	Brain	Bulk	GSE: https://www.ncbi.nlm.nih.gov/geo/query/acc.cgi?acc=GSE114129	Xu et al., (2018)
*Heterocephalus glaber*	4 years old (*n* = 1), 20-years old (*n* = 1)	Brain, Liver, Kidney	Bulk	GSE: https://www.ncbi.nlm.nih.gov/geo/query/acc.cgi?acc=GSE30337	Kim et al., (2011)
*Mus musculus*	4 mo-old (*n* = 3), 24 months old (*n* = 3)	Astrocyte enriched from cerebellum, hypothalamus, motor cortex, and visual cortex	Bulk	GSE: https://www.ncbi.nlm.nih.gov/geo/query/acc.cgi?acc=GSE99791	Boisvert et al., (2018)
*Mus musculus*	3-months-old^b^, 18-months-old^b^, 24-months-old^b^	Lung, Liver, Muscle, Brain, Skin, Kidney	Single cell	GSE: https://www.ncbi.nlm.nih.gov/geo/query/acc.cgi?acc=GSE132042	Tabula Muris Consortium, (2020)

Age groups and tissue sample sizes refer to only those individuals analyzed within this study, i.e. healthy adults (no drug treatment, etc.).

^a^Each sample represents a pool of 18 individuals.

^b^Detailed sample size information is given in Table S3.

**Fig. 1.**
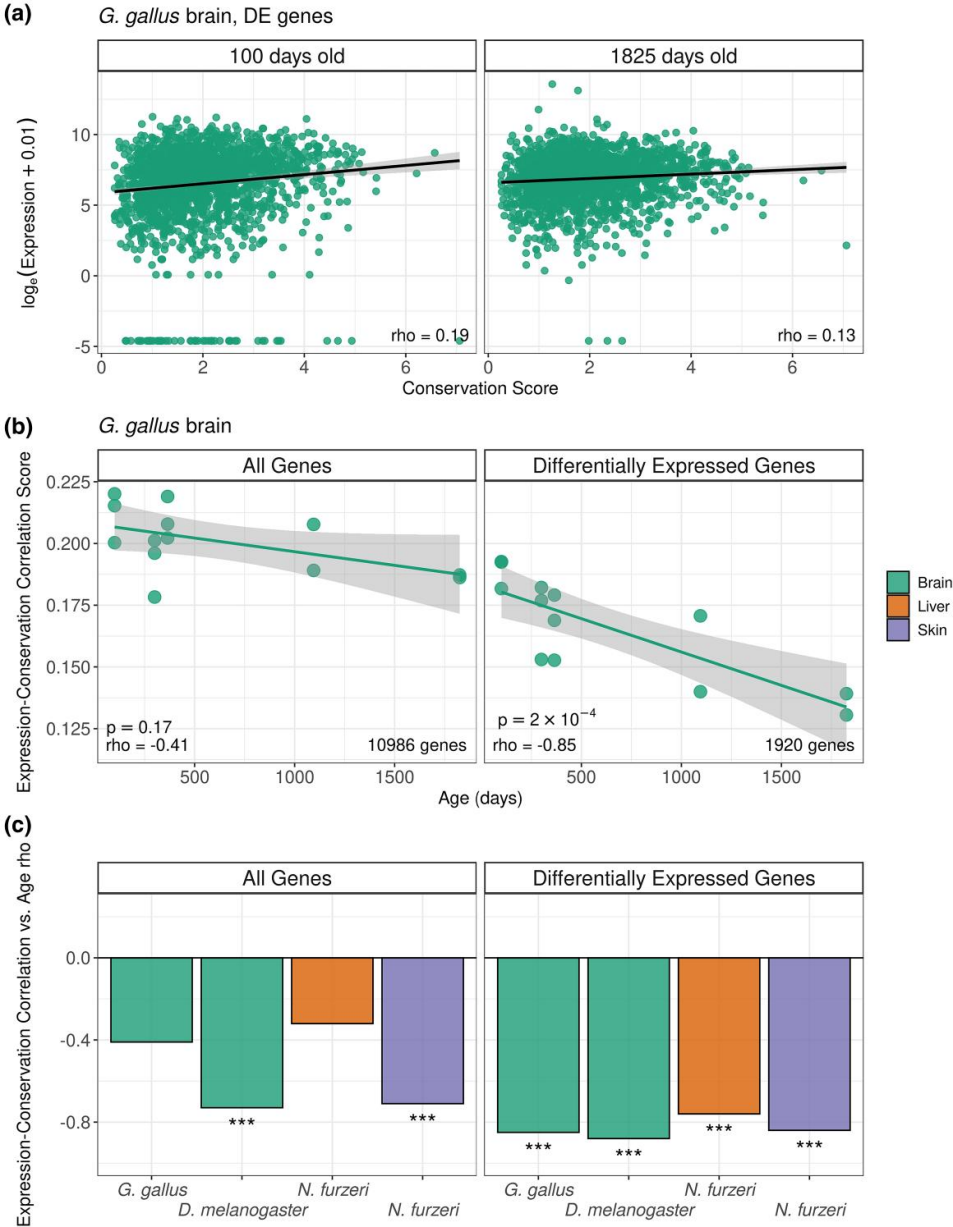
Age-related changes in expression-conservation correlation in diverse metazoa. (a) Expression-conservation correlations for genes differentially expressed in ageing in the brains of two exemplary individuals of *Gallus gallus*, aged 100 and 1,895 days respectively. *rho* values in the inset represent the Spearman's correlation values between expression and conservation scores of genes. (b) Age-related changes in expression-conservation correlation in the *G. gallus* brain. The *y*-axis shows the Spearman correlation coefficient between expression level and the protein sequence conservation metric across genes for each individual in this dataset. Each dot corresponds to an individual (*n* = 13). The *x*-axis shows the individual age. The left panel shows the analysis result using the whole transcriptome (*n* = 10,986), and the right panel shows the result using the genes differentially expressed with age (*n* = 1,920). Spearman correlation *rho* and *P*-values in the inset are calculated between the expression-conservation correlation and individual age. (c) Summary of Spearman correlations between expression-conservation and age for different dataset, (***): *P* < 0.001. Tissues are illustrated in different colors (key shown on the right hand side). Differentially expressed genes were chosen using a cut-off of |*rho*| > 0.5 (see Fig. S3 for alternative cut-offs).

Using the same framework used by Turan and colleagues (Turan et al. 2019), we calculated the correlation between protein sequence conservation metrics (based on *dN*/*dS*; see Methods) and expression levels across genes for each individual. We used this correlation value as a metric of transcriptome conservation per sample, which integrates expression levels of genes in the transcriptome with conservation scores per gene, creating an expression level-based transcriptome conservation metric. We then investigated how transcriptome conservation levels change with age by studying the relationship between the expression-conservation correlation scores and individual age (Fig. 1a and b, showing the *G. gallus* brain dataset as example). This expression-conservation-age correlation approach uses information across age-series and thus can be more powerful relative to the standard approach of dividing genes into young- and old-biased classes.

The results for the whole transcriptome (i.e. using all expressed genes) revealed moderate ADICT patterns in all four datasets, as reflected in negative expression-conservation-age correlations (Fig. 1b, summarized in Table S5). The ADICT signal became more conspicuous when we limited the analysis to differentially expressed genes (Fig. 1a–c). We additionally used alternative expression-age correlation coefficient (*rho*) cut-offs for classification of differentially expressed genes, which showed our results did not depend on cut-off choice (Fig. S3).

**Fig. 2.**
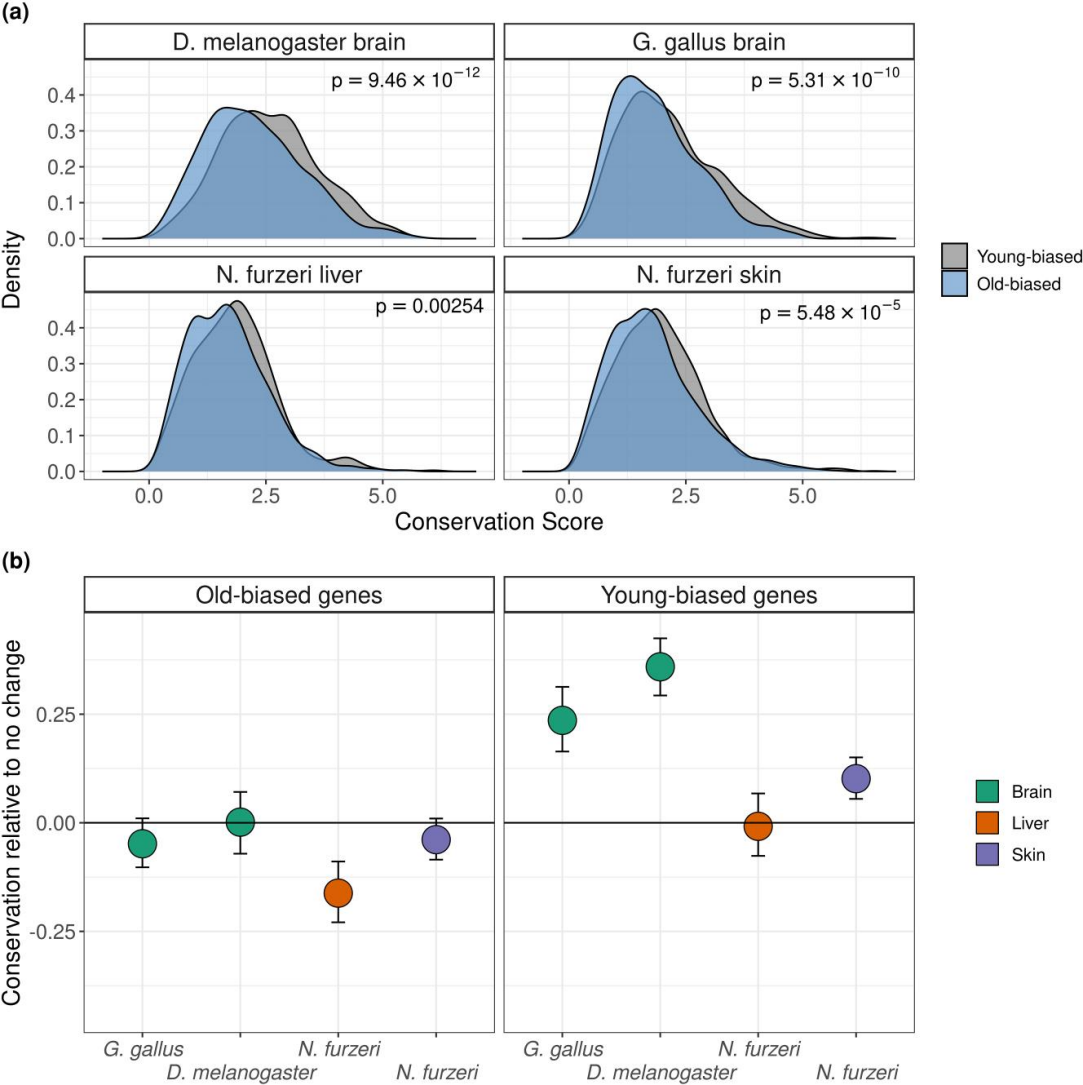
Sequence conservation differences between old-biased and young-biased genes. (a) Density plots of conservation scores of old- and young-biased genes, P-values indicate the results of Welch's *t*-test (two-sided) between the distributions of old-biased and young-biased genes. (b) Mean conservation scores of old-biased and young-biased genes, relative to constantly expressed genes. Error bars indicate 95% confidence intervals calculated using 1,000 bootstraps (*D. melanogaster* brain *n*_old-b_ = 792 genes, *n*_young-b_ = 831 genes; *G. gallus* brain *n*_old-b_ = 1,066 genes, *n*_young-b_ = 864 genes; *N. furzeri* skin *n*_old-b_ = 1,468 genes, *n*_young-b_ = 1,405 genes; *N. furzeri* liver *n*_old-b_ = 537 genes, *n*_young-b_ = 618 genes).

ADICT could be caused by highly conserved genes decreasing in expression with age or by weakly conserved genes increasing in expression with age, or both. To test this, we examined gene-wise conservation levels of young-biased genes (that show significant negative expression vs. age correlation with *rho* < -0.5) and old-biased genes (that show significant positive expression vs. age correlation with *rho* > 0.5) for each data set. Old-biased gene sets showed consistently lower average gene-wise conservation than young-biased genes in all four datasets (Welch's t-test *P* < 0.003 across all four tests, Fig. 2a, Fig. S4). Old-biased and young-biased gene sets also respectively showed trends of lower and higher gene-wise conservation compared to constantly expressed genes (Fig. 2b). Turan and colleagues had also observed similar trends, with some degree of variability across mammalian tissues (Turan et al. 2019).

Next, we asked whether the observed patterns may be simply explained by differences in gene age and mean expression level between young- and old-biased genes, which are known to correlate with gene conservation (Albà and Castresana 2005; Drummond et al. 2005). To address this we used linear models that incorporate gene age, average/maximum expression and gene class (young- vs. old-biased) as explanatory variables to explain variation in *dN/dS* across genes (see Methods). The results consistently showed gene class as a statistically significant explanatory variable of gene conservation when other factors are accounted for (Table S8), indicating that age-related expression is related with conservation independent of gene age and mean expression level.

In addition, we analyzed published transcriptome data from naked-mole rats *H. glaber*, an exceptionally long-lived eusocial rodent, which can survive more than 30 yr in captivity (Kim et al. 2011). Both breeding and nonbreeding naked-mole rats were recently reported not to show increasing age-specific hazard of mortality (Ruby et al. 2018), consistent with the case of long-lived ant queens described earlier (Harrison et al. 2021). The dataset consists of tissue samples from the brain, kidney and liver of a single 4-year-old and another 20-year-old female naked-mole rat. Consequently, we could only classify genes into old-biased and young-biased groups based on expression trends (i.e. we could not use expression-conservation-age correlations). In all three tissues, old-biased genes showed weaker gene-wise conservation than young-biased genes. Nevertheless, we mark that the lack of biological replicates does not allow generalizing the result (Fig. S5).

### Cell Type Transcriptomes Vary Dramatically in Average Conservation Levels

ADICT patterns (lower conservation of old-biased genes) we observed on bulk-tissue transcriptomes could be driven by (i) cell type composition changes, such that cell types with weakly conserved transcriptomes become more abundant in tissues or (ii) cell type-specific changes, such that old-biased genes in each specific cell type tends to be weakly conserved, or both. Notably, scenario (i) assumes the presence of significant differences among specific cell types with respect to average conservation levels of their transcriptomes. To investigate such possible conservation differences among cell types, we used published single-cell transcriptome data from 44 cell types across lung, skeletal muscle, brain, skin, and kidney (Tabula Muris Consortium 2020). We estimated a transcriptome conservation level for each cell type, calculated as the correlation between conservation scores and the mean gene expression levels of all cells assigned to that cell type in a given individual, using young adult mice, and using the combined set of expressed genes across cell types (*n* = 13,989) (Methods). We note that we are not studying an age effect here, but only transcriptome conservation differences between cell types. We also note that because we use the same set of genes for all cell types, differences in the correlation coefficient represents average conservation differences among cell types.

**Fig. 3.**
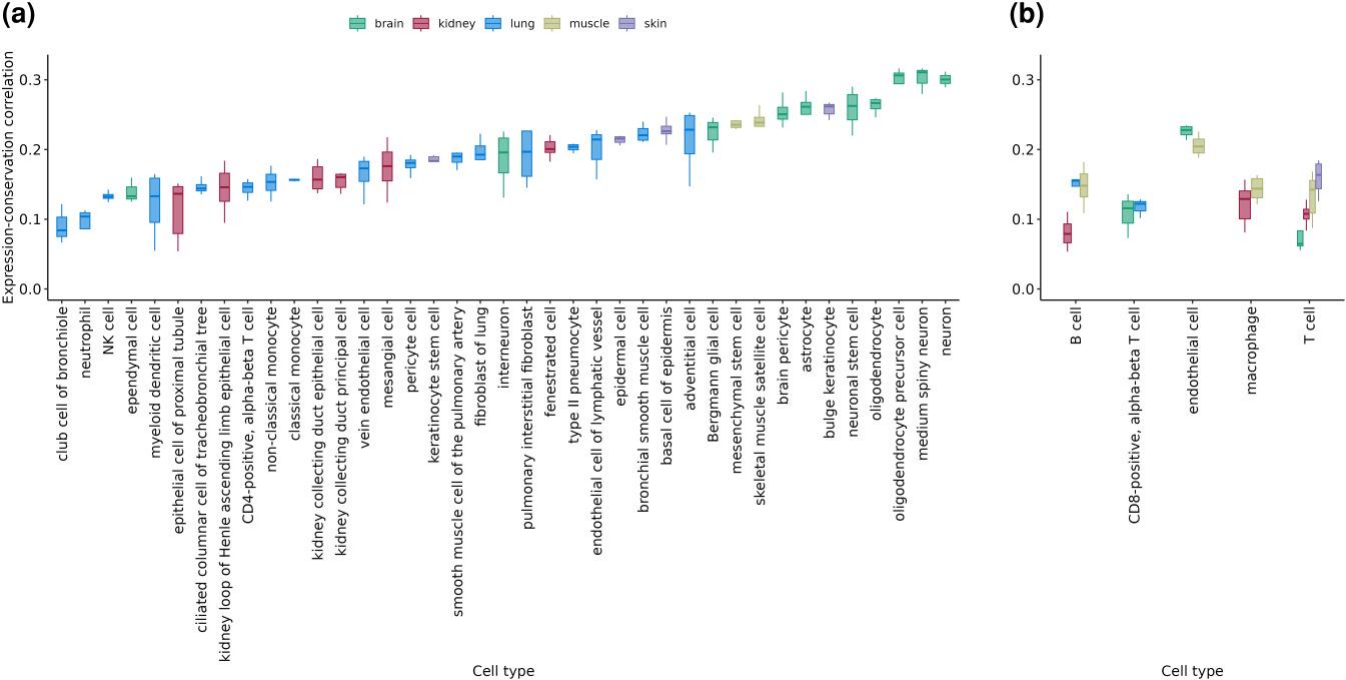
Variability of expression-conservation correlation levels across cell-type transcriptomes of young-adult (3-month-old) individuals (Tabula Muris Consortium 2020). Each data point within boxplots represents Spearman's correlation values for an individual, calculated using gene expression values averaged across cells belonging to that individual. Panels a and b show cell types sampled in only one tissue and cell types sampled from multiple tissues, respectively. Color-coding indicates the tissues which the cell types were sampled from (see key at the top of the figure). Cell types with less than three correlation values (i.e. individuals) are excluded.

The results shown in Fig. 3 demonstrate salient variation among specific cell types in their average transcriptome conservation levels. There was a significant cell type effect along with a tissue effect in a two-way ANOVA (*F*_tissue_ = 89.1, d.f._tissue_ = 4, *F*_celltype_ = 17.8, d.f._celltype_ = 43, *P* < 1 × 10^−16^ for both factors; Table S6). In general, neural cells, oligodendrocytes and astrocytes showed the highest transcriptome conservation, parallel to earlier observations indicating high protein sequence conservation of brain-specific genes (Khaitovich et al. 2005). Certain specialized and proliferative cells in other tissue types, such as keratinocytes, skeletal muscle satellite cells and mesenchymal stem cells also showed higher-than-average transcriptome conservation. Immune cells showed the opposite trend, with various types (including B-cells, T-cells, neutrophils, myeloid dendritic cells) on the lowest end of the transcriptome conservation distribution in our sample. A mixed model ANOVA with immune status as the explanatory variable and tissue as random effect also revealed a negative significant effect of immune status on transcriptome conservation (*F*_immune status_ = 95.79, d.f. = 1, *P* < 0.0001). However, immune cells were not alone in low transcriptome conservation patterns; some tissue-specific cell types, such as the epithelial cell of the proximal tubule (kidney) and the club cell of the bronchiole (lung) also displayed low transcriptome conservation. We also repeated the same analysis using cell type transcriptomes of 18-month-old and 24-month-old mice, which revealed similar results (Fig. S6).

### Cell Type-specific Changes in Transcriptome Conservation With Age

**Fig. 4.**
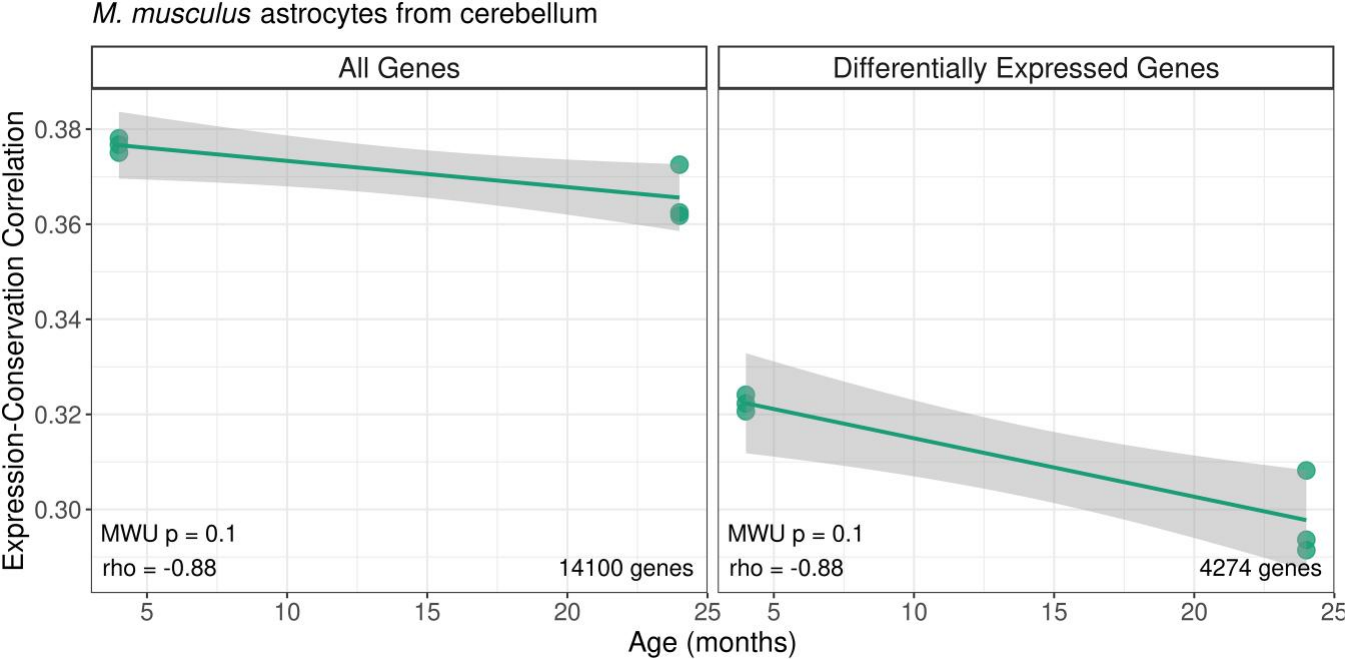
Age-related changes in expression-conservation correlation in *M. musculus* astrocyte transcriptome from the cerebellum. The *y*-axis shows the Spearman correlation coefficient between expression level and protein sequence conservation metric across genes for each individual in this dataset (*n* = 6). The *x*-axis shows individual age. The left panel shows the analysis results using the whole transcriptome (*n* = 14,100), and the right panel shows the results for genes differentially expressed with age (*n* = 4,274). *rho* values in the inset indicate the results of the Spearman correlation between the expression-conservation correlation and age, while *P*-values indicate the results of the Mann–Whitney U tests.

The strong variation in transcriptome conservation observed among mammalian cell types raises the possibility that cell type composition changes might be the sole driver behind ADICT. If true, we should observe no ADICT signal *within* cell type-specific transcriptomes. To address this, we leveraged upon two cell type-specific ageing transcriptome datasets. The first was an ageing transcriptome dataset (Table 1) consisting of young (4-month-old, *n* = 3) and old (24-month-old, *n* = 3) mouse tissue samples from cerebellum, hypothalamus, motor cortex, and visual cortex; these were enriched for astrocytes using astrocyte-ribotagging, which can differentially capture actively translated portions of the transcriptome from tagged astrocytes (Boisvert et al. 2018). From four different brain regions, we identified significant age-related expression changes (see Methods) in the cerebellum and hypothalamus, which we investigated further. Analyzing the data using the same expression-conservation-age correlation framework described earlier, we found a negative signal using differentially expressed genes in astrocyte transcriptomes of the cerebellum and hypothalamus, indicating ADICT (Fig. 4 and Fig. S7; Table S5).

**Fig. 5.**
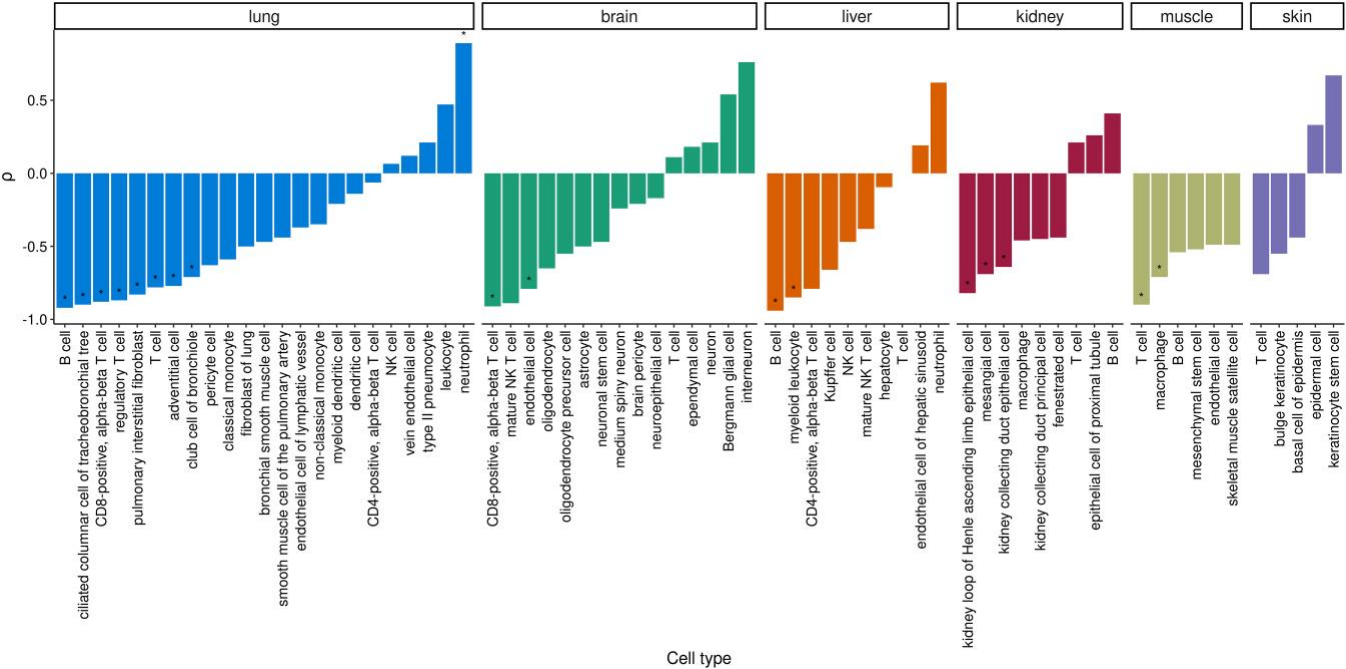
Spearman correlation results for expression-conservation correlation (for differentially expressed genes) versus age in six different tissues from the Tabula Muris Senis dataset. (*) marks indicate statistical significance at q-value <0.1 (BH corrected Spearman correlation test *P*-value).

We next analyzed single-cell transcriptomes from the Tabula Muris Senis dataset, comprising a total of 23,538 cells across 53 cell types from 14 different individuals, covering 3-months, 18-months and 24-months of age (*n*_lung_ = 12, *n*_liver_ = 7, *n*_muscle_ = 14, *n*_brain_ = 9, *n*_skin_ = 10, *n*_kidney_ = 14) (Tabula Muris Consortium 2020) (see Table S3 for detailed sample sizes). Among the cell types that are not shared between tissues, 32 out of 44 showed the ADICT pattern, i.e. negative expression-conservation-age correlations, of which six cell types were significant at *q* < 0.05 (Spearman correlation test with multiple testing correction), and eight at *q* < 0.1. Among the nine cell types that were detected in more than one tissue, five (CD4-positive alpha-beta T cell, CD8-positive alpha-beta T cell, mature NK T cell, macrophage, and endothelial cell) showed consistent ADICT signal across the tissues, three (T cell, NK cell, and B cell) showed inconsistent signals, while one (neutrophil) showed a positive correlation (Fig. 5). Only in the skin did no cell type reach the *q* < 0.1 threshold (Fig. 5). Across all tissues, only one cell type, lung neutrophil, showed positive correlation that reached statistical significance at *q* < 0.1 (Figure 5, Table S7).

**Fig. 6.**
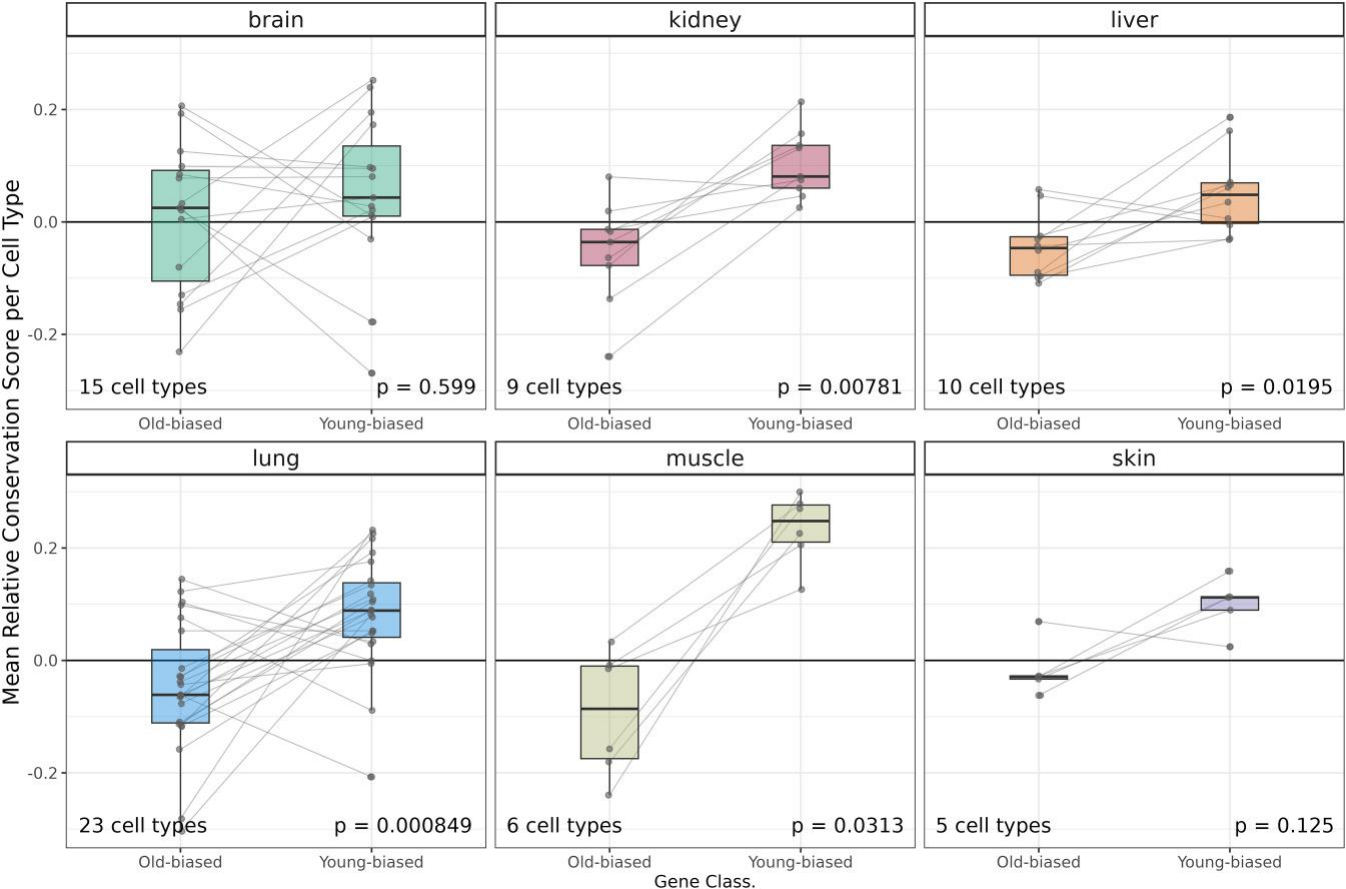
Conservation differences between old- and young-biased genes per cell type. The *y*-axis shows the mean relative conservation score (relative to constantly expressed genes; see Methods) for old-biased and young-biased genes in each cell type, in each tissue (sample sizes for cell types are *n*_brain_ = 15, *n*_kidney_ = 9, *n*_liver_ = 10, *n*_lung_ = 23, *n*_muscle_ = 6, *n*_skin_ = 5 respectively). The lines connect the mean relative conservation scores calculated for old-biased and young-biased gene sets for the same cell type. The *P*-values indicate Wilcoxon signed rank test results for the difference between the distribution of per cell type mean conservation scores of old-biased and young-biased genes in that tissue (no correction for multiple testing).

We further compared average gene-wise conservation levels between old-biased and young-biased gene sets identified in each of the cell types separately for each tissue. Across cell types in all six tissues we observed a trend toward lower gene-wise conservation among old-biased genes relative to young-biased genes identified in each cell type; this was significant in four tissues (two-sided Wilcoxon signed rank test *P* < 0.05) (Fig. 6). ADICT can thus be observed at the cell type-specific transcriptome level, at least in the mouse and for a substantial number of cell types. Meanwhile, the signal is heterogeneous both among tissues and also among cell types within a tissue.

### ADICT Propensities of Immune Versus Nonimmune Cells

**Fig. 7.**
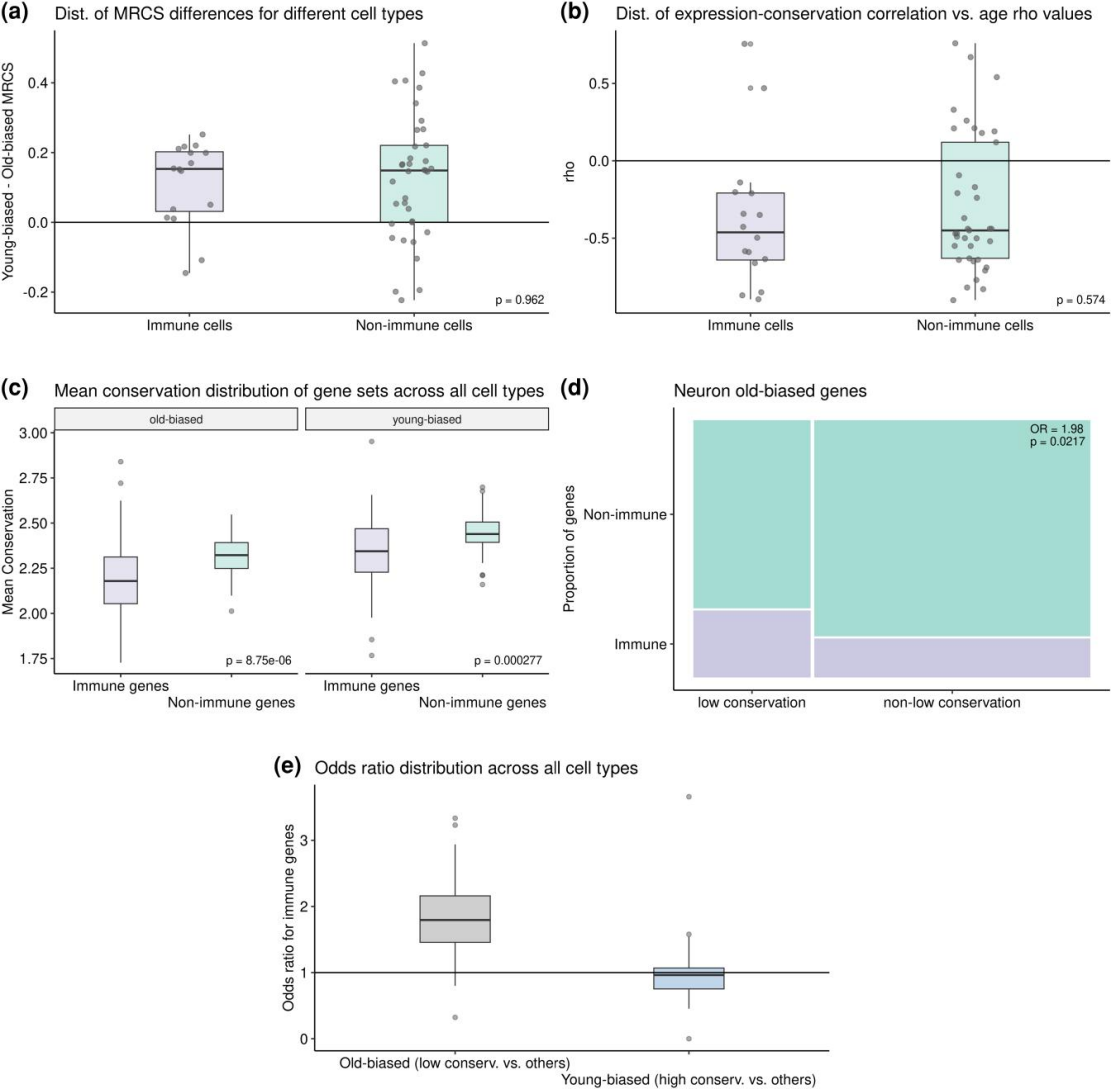
Distributions of (a) mean relative conservation score (MRCS) differences between young-biased and old-biased genes and (b) spearman's correlation values for expression-conservation correlation versus age for immune and nonimmune cell types. (c) Distribution of mean conservation scores between immune and nonimmune gene sets for both old- and young-biased gene groups among all cell types. (d) Proportion of immune and nonimmune genes for low conservation and non-low conservation genes among old-biased genes in neuron data, shown as an example. (e) Distribution of odds ratios for immune overrepresentation in old-biased (low conservation and other genes comparison) and young-biased (high-conservation and other genes comparison) gene sets for all 53 unique cell types and 68 individual data points across six tissues. P-values for A, B, and C panels indicate results of Mann–Whitney U tests and *P*-value for the D panel represents the result of Fisher's exact test.

Dysregulated immune responses and inflammation are among the most common hallmarks of ageing (López-Otín et al. 2013). Ageing-related chronic inflammation is known to result in an increase in immune cell types within inflamed tissues. Immune cell types were ubiquitous across the mouse tissues we analyzed in this study. Based on our results that genes in the transcriptomes of some immune cell types show a pattern of lower conservation compared to other cells (see Fig. 3), we further asked whether stronger ADICT propensity among immune cell types might be a contributor of ADICT patterns at the bulk-tissue level. Specifically, we tested whether immune cell types show a difference in ADICT propensity compared to other cell types using 16 immune and 37 nonimmune cell types from the Tabula Muris Senis dataset (Table S4). For this, (i) we compared ADICT signals between immune and nonimmune cell types using expression-conservation-age correlations, and (ii) calculated the mean relative conservation score (MRCS) differences between young-biased and old-biased genes for immune and nonimmune cell types, and compared the two (Methods). Neither analyses revealed a significant difference between immune and nonimmune cell types [Mann–Whitney *U* test *P*-values = 0.96 and 0.57, Cohen's *d* = 0.195 and 0.107 for (i) and (ii), respectively] (Fig. 7). These results suggest that ADICT is not solely caused by immune cells with lowly conserved transcriptomes being more represented in old individuals' tissues.

We further investigated the possible contribution of immune-related genes to ADICT patterns by comparing mean conservation scores of old- and young-biased immune versus nonimmune gene sets for all of the cell types. This revealed that immune-related genes, as expected, are significantly less conserved on average than nonimmune genes; this was valid for both old- and young-biased gene sets (Fig. 7c). We then asked whether immune-related genes may be overrepresented in gene sets that could contribute to ADICT pattern: i) old-biased and low-conserved, and ii) young-biased and high-conserved gene sets. Among old-biased genes, the low-conserved group contains on average 1.84 times more immune-related genes compared to the rest of the old-biased genes, with 27 of the 53 cell types having a significant *P*-value (Fisher's exact test) (Table S9). Figure 7d shows an example for neurons, while Fig. 7e shows the distribution of odds ratios across the 53 cell types. Interestingly, we did not see such a trend for young-biased genes (Fig. 7e). These results suggest that immune genes might be a major contributor to the ADICT pattern among old-biased genes.

### ADICT at the Population Level

In the above analyses, we studied gene-wise evolutionary conservation using inter-species divergence data, which capture the accumulated impact of purifying selection in the long-term. This can be more powerful than estimates of evolutionary conservation based on intra-species polymorphism, because the latter reflects the impact of negative selection only in the short-term. Still, work on human data within the last decade has suggested that age-related variation in purifying selection can shape functional polymorphism levels (Rodríguez et al. 2017). This motivated us to study the conservation difference between old-biased and young-biased genes at the population level in mice. For this, we utilized publicly available population genetics data from seven different wild populations of house mice from North America and Europe, and calculated gene-wise Tajima's D scores, where negative values indicate an excess of singletons, which can be caused by purifying selection. We then compared young-biased and old-biased genes identified in the mice astrocyte dataset, which revealed a modest difference, with young-biased genes showing a trend of lower Tajima's D values (i.e. more singletons) than old-biased genes (Fig. S8). Using young-biased and old-biased genes calculated using cerebellum and hypothalamus data and Tajima's D values calculated using combining all populations, we found a statistically significant difference (Mann–Whitney *U* test *P*-values < 0.05) in both regions (Fig. S8). We also observed statistically significant lower values in young-biased genes for Florida, New Hampshire/Vermont, and Virginia populations for cerebellum and Florida population for hypothalamus, with the rest of the comparisons not reaching statistical significance (Fig. S8). This suggests that the ADICT effect can also be captured at the population level, although the signal is weaker compared to using inter-species conservation metrics.

## Discussion

Our results indicate ADICT as a common phenomenon across diverse metazoan taxa with classical senescence patterns, i.e. increased mortality with age. Lower conservation of old-biased genes was previously reported in primate and rodent tissues (Somel et al. 2010; Jia et al. 2018; Turan et al. 2019), as well as whole-body transcriptomes of Diptera (Cheng and Kirkpatrick 2021). We now add birds and fruit flies to the list of taxa in which ADICT has been observed at the tissue level. Interestingly, we also observe low sequence conservation among old-biased genes in three tissues of the extreme long-lived naked-mole rat, even though the lack of biological replication in this dataset means that this result is only tentative. Together, our findings support a widespread role of the selection shadow, or drift, in shaping organismal senescence.

We further show that average transcriptome conservation levels are highly variable between different cell types, at least in the mouse. This result echoes observations on varying conservation levels among mammalian tissues (Khaitovich et al. 2005; Kryuchkova-Mostacci and Robinson-Rechavi 2015). Neurons show highest transcriptome conservation among the 44 mouse cell types analyzed here, which parallels strong conservation of brain-expressed genes observed in these earlier studies, possibly due to sensitivity of neural cells to proteotoxicity (Drummond and Wilke 2008). Immune cells tend to show weak conservation, which may be partly attributable to the relatively high rates of positive selection pressure on immune-related genes [e.g. The Chimpanzee Sequencing and Analysis Consortium 2005]. Although these results are not surprising, they point to the difficulty in interpreting ageing-related transcriptome changes and ADICT signals at the bulk tissue and/or whole body level, given ageing-related changes in cell type composition with age [e.g. (Tabula Muris Consortium 2020)].

Our third main finding is that ADICT may prevail at the cell type level at least as widely as at the bulk-tissue level. We show this using enriched astrocyte transcriptomes, as well as single-cell RNA sequencing data from six tissues in mice. Importantly, here we are assuming that cell types are sufficiently homogeneous, their identities are accurately defined, and these characteristics are not affected by ageing. With this note of caution, and the fact that we have analyzed single-cell data from only a single species, our results suggest that previously identified ADICT patterns are not solely driven by cell type composition changes. In addition, we find that even though immune-related genes contribute to ADICT, the ADICT pattern is not exclusive to immune cells. We thus believe that the evidence we present here, together with earlier work, marks a major presence of ADICT in metazoan ageing. However, two main questions remain.

The first involves the causal role of ADICT in organismal senescence. In other words, does the high expression of low-conserved genes compromise fitness? This can be addressed by selection experiments, by comparing species pairs which have recently evolved differences in lifespan, as well as by studying species with constant or decreasing mortality with age (Jones et al. 2014; Cohen 2018). We predict that the evolution of a constant or decreasing mortality curve, or longevity, should lead to increasing purifying selection on old-biased genes, which should be measurable using standard transcriptome experiments.

This has been exactly what Harrison and colleagues have observed: higher protein sequence conservation among old-biased genes in ant queens (Harrison et al. 2021). However, it remains possible that age-related changes in cell type composition (e.g. increasing proportion of reproductive tissue) could underlie the observed signal in that study. Intriguingly, three tissues of the naked-mole rat that we analyze here show expression patterns consistent with ADICT, even though this dataset suffers from lack of biological replication. We believe that continuing this line of work with improved sampling could be highly illuminating with respect to the causal role of mutation accumulation in the evolution of ageing. We also note that the presence of ADICT in model organisms in ageing research, including the mouse, killifish and fruit fly, opens up the possibility of studying ADICT in longevity selection experiments.

The second question relates to the nature of old-biased and low-conserved genes, which needs systematic dissection. This is especially interesting in light of the results by Jia and colleagues (Jia et al. 2018) and by Cheng and Kirkpatrick (Cheng and Kirkpatrick 2021), who showed that old-biased genes tend also to be evolutionarily younger genes. This brings up the possibility of two distinct hypothetical mechanisms behind ADICT:

1. ADICT by regulatory drift: Part of metazoan proteomes consists of genes that have relatively recently evolved, are expressed at low levels, and which may be less optimized in their structure and functions, including a propensity toward proteotoxicity due to mistranslation, misfolding, or misinteraction. Suboptimal splicing regulation could also be a contributor to such effects, a possible topic for future work. Genetic drift on regulatory sequences and regulatory interactions may drive late-expression of such low-conserved genes, while their early expression would not be tolerated. Recruitment of such genes in late adult transcriptomes could drive ADICT and shape the ageing phenotype. This would be in line with “early-life inertia” model of ageing (e.g. de Magalhães and Church 2005; Carlsson et al. 2021), as well as empirical evidence for faster evolution of promoter sequences in old-biased genes in mammals (Turan et al. 2019).
2. ADICT by drift on coding sequences: Old-biased genes may have critical roles at old age (e.g. protection against accumulating somatic damage). Therefore, being old-biased may be a shared property of genes across different species. However, drift on their coding sequences may prevent them from functioning at the highest efficiency and/or lead to mutations that may also cause mistranslation, misfolding or misinteraction (see Drummond and Wilke 2008; Pérez et al. 2009; Schneider et al. 2018; Llewellyn et al. 2024)]. Amino acid changes that promote protein stability have been observed to evolve convergently in long-lived mammals (Farré et al. 2021), which would support the idea that the accumulation of suboptimal coding variants in the gene pool and their expression in old-biased genes could cause somatic damage accumulation and ageing phenotypes. This model could be tested by comparing the strength of selection on old-biased genes in the genomes of closely related short- and long-lives species using tools such as RELAX (Wertheim et al. 2015). For instance, Cui and colleagues showed genome-wide signals of relaxation of selection in killifish that recently evolved short lifespans (Cui et al. 2019); conversely, we would predict higher selection on old-biased genes when there occurs a shift toward increased longevity.

Noting that the two ADICT models are not mutually exclusive, we believe that uncovering their exact contributions to the selection shadow in ageing would be an attractive endeavor.

## Materials and Methods

### Conservation Score

When available, *dN* (nonsynonymous substitution rate) and *dS* (synonymous substitution rate) values were downloaded from the Ensembl BioMart using the most recent Ensembl releases that included these metrics (Ensembl v.99 for *dN* and *dS* values between *G.gallus*-*M.gallopavo* and *M.musculus*-*R.norvegicus*; Ensembl Metazoa v.45 for *dN* and *dS* values between *D.melanogaster*-*D.simulans*) (Yates et al. 2019). Only one-to-one orthologs, estimated by Ensembl, were included in the study. The conservation metric was calculated as *–ln(dN/dS)*, with higher values corresponding to higher sequence conservation. Genes with *dN/dS* ≥ 0.8 were excluded to limit the influence of positively selected genes on downstream analysis. Genes with *dN* = 0 and *dS* = 0 were also excluded to avoid zero and infinite *dN/dS* values, respectively. *dN* and *dS* values for killifish gene orthologs were not available in BioMart and we used *dN/dS* values calculated for the “FKK-branch” by Sahm and colleagues (Sahm et al. 2017). Statistics related to the conservation metric data (mean and median *dN/dS* values, gene numbers, divergence between species pairs) are provided in Table S1. We also ignored genes with paralogs in this analysis in order to limit uncertainty and ambiguity when calculating dN/dS ratios.

We note that the vast majority of genes (83% to 98%, see Table S1) in these datasets have *dN/dS* values <0.8, which is consistent with being functional and evolving under purifying selection. The conservation score we calculate below captures the relative degree of purifying selection strength across these genes.

### Relative Conservation Score

To simplify interpreting and comparing the conservation score values when visualizing, we scaled these using the conservation scores of constantly expressed genes (defined below). For this, in each dataset, we calculated a “relative conservation score” for each gene *i* as:


$${\mathrm{RCS}}_{i}={\mathrm{CS}}_{i}-{\mathrm{MCS}}_{\mathrm{constant}}$$

where *CS_i_* is the protein sequence conservation score for that gene (as described above), and *MCS*_constant_ is the arithmetic mean conservation score for all genes classified as constantly expressed in that dataset (explained below).

## Bulk RNA-Seq Data Analyses

### Preprocessing

Raw FASTQ files for the mice brain (SRP108790, GSE99791) (Boisvert et al. 2018), chicken brain (SRP144776, GSE114129) (Xu et al. 2018), and naked-mole-rat brain (SRP007398, GSE30337) (Kim et al. 2011) transcriptomes were downloaded from the European Nucleotide Archive (ENA) repository. The dataset contents are listed in Table 1. FASTQ files were assessed for quality using *FastQC* tool (v0.11.9) and trimmed for low quality and adapter contamination using *Trimmomatic* (v0.39) (Bolger et al. 2014) with the options *SLIDINGWINDOW:4:15*, *MINLEN:25* & *<adapter fasta>:2:30:10:8:TRUE*. Trimmed reads were aligned to the reference genome (chicken GRCg6a, mouse GRCm38.p6, naked-mole rat HetGla_female_1.0) and counted using *STAR* (v2.7.6a) (Dobin et al. 2013) with the parameter *–quantMode GeneCounts*. If present, drug-treated and nonadult samples were discarded from the datasets, along with genes not expressed in any of the remaining samples in each dataset. Resulting count data were normalized using the median ratios method implemented in the DESeq2 (v1.26.0; Love et al. 2014) package. Briefy, we used the *DESeqDataSetFromMatrix* function from the DESeq2 package, with the arguments “*design*  *=*  *∼age”* to construct a DESeqDataSet object, and used the *estimateSizeFactors* function to estimate size factors for normalizing raw counts. For the killifish liver and skin transcriptomes (Reichwald et al. 2015) and the fruit fly brain transcriptomes (Pacifico et al. 2018), gene count data were used instead of the raw FASTQ files. Count data for these datasets were normalized as before. We studied the preprocessed datasets by performing principal components analyses and plotting the first four principal components (Figs. S1 and S2).

### Differential Gene Expression Analysis

Genes that show ageing-related expression changes were identified using Spearman correlation: a gene was classified as age-related if the expression of the gene showed a statistically significant correlation with age after multiple testing correction (Benjamini-Hochberg corrected *P* < 0.1), and an absolute value of expression versus age correlation coefficient (*rho*) higher than 0.5. We additionally tested the sensitivity of our analyses to the choice of *rho* by using alternative cut-offs of 0.3 and 0.7 (Fig. S3). Note that we only used data from adult individuals and excluded preadults (Table 1). Among age-related genes, genes with positive expression-age correlation were classified as “old-biased”, and the genes with negative expression-age correlation were classified as “young-biased”. Genes outside either class were classified as “constantly expressed”. Genes assigned to each gene class are shown in Table S2. For the naked-mole-rat, due to lack of biological replicates in this dataset, genes with positive Spearman correlations between expression versus age were classified as old-biased genes and the ones with negative correlation were classified as young-biased genes, without reference to statistical significance.

### Transcriptome Conservation

Here we followed the approach outlined by Turan and colleagues (Turan et al. 2019). For each individual sample in a dataset, we calculated a single expression-conservation correlation measure, reflecting the correlation between normalized gene expression values and the conservation metric across all genes, using the Spearman correlation test via the *cor* function in *R* (v. 3.6.3) *stats* package, with the arguments “*method*  *=*  *spearman”*. We then calculated the correlation between individual age and expression-conservation correlations across all individuals in a dataset, again using Spearman correlation. This latter correlation was treated as a measure of age-related change in overall transcriptome conservation (Fig. 2).

### Conservation Difference Between Old-biased and Young-biased Genes

To test whether the distribution of conservation scores differs between old-biased and young-biased genes we used the Welch's t-test (two sided) on conservation scores of old and young-biased genes separately for each tissue in each dataset, as implemented by the *t.test* function in the *stats* package of *R* with default parameters. As a nonparametric alternative, we also used the Mann–Whitney *U* test as implemented by the *wilcox.test* function in the *R stats* package, using default parameters.

### Controlling for Gene Age and Expression

To be able to compare expression values between different genes in a given dataset, we calculated TPM values per transcript for *Gallus gallus* brain and *Mus musculus* astrocyte datasets using kallisto v.0.50.0 (Bray et al. 2016) with default parameters. We then summed up TPM values of all transcripts that belong to a gene to come up with gene-wise expression values. Using gene age data obtained from GenOrigin (Tong et al. 2021), mean/maximum expression values and gene class information, we constructed multiple linear regression models to test the statistical significance of those variables in effecting dN/dS values using base R function lm() with the parameters “formula = dnds ∼ mean_exp + gene_age + gene_class” and “formula = dnds ∼ max_expd + gene_age + gene_class”.

### Tajima's D

We used a publicly available autosomal SNP variation dataset of *Mus musculus* from seven different populations across Eastern USA (Florida, Georgia, New Hampshire/Vermont, Pennsylvania, Virginia) and Western Europe (France, Germany) (Agwamba and Nachman 2023, originally published in Harr et al. 2016; Phifer-Rixey et al. 2018). The original VCF was split into different populations using BCFTools v.1.18 (Danecek et al. 2021). We then used VCF-kit v.0.2.9 (Cook and Andersen 2017) with the arguments “*tajima 10000 10000*” to calculate Tajima's D scores across the genome in 10 kb long windows. Gene-wise Tajima's D scores were calculated by taking the weighted mean of the windows that the genes of interest fell into, by mapping the output of VCF-kit to coding sequences in the reference genome GRCm38.p6 using BEDOPS v2.4.41 (Neph et al. 2012) *bedmap*. We compared Tajima's D scores of genes that were categorized as old-biased and young-biased in the mice astrocyte dataset using the Mann–Whitney *U* test as implemented in R *wilcox.test* function with default parameters.

## Single-cell RNA-Seq Data Analyses

### Preprocessing

Single-cell expression data for six different tissues (lung, liver, muscle, brain, skin, and kidney) were downloaded from the Tabula Muris Senis dataset (Tabula Muris Consortium 2020) and processed following Izgi and co-authors (Izgi et al. 2022). For each cell type, the gene expression levels per individual were calculated as the mean expression value across cells of that cell type from a given individual, calculated separately for each gene, and separately in each tissue. We removed cell types absent in any of the three age groups (3-month-old, 18-month-old, 24-month-old). To minimize the individual effect in downstream analyses, we limited our analysis to include only individuals that had expression data for >70% of all cell types present within a given tissue for a given age group (irrespective of the number of cells measured for that cell type). The final number of cell types and individuals are presented in Table S3.

### Cell Type-Specific Differences in Transcriptome Conservation

To evaluate the cell type-specific differences in transcriptome conservation, in each tissue we calculated the Spearman correlation coefficient between expression levels and conservation scores as above, only using the three-months old individuals for every cell type. To be able to compare expression-conservation correlation across different cell types, we used the common gene set of genes expressed across all tissues (*n* = 13,989). We excluded cell types represented by less than three individuals. We then tested the cell type difference in expression-conservation correlation using ANOVA as implemented in the *aov* function in the *R stats* package with the model “*rho ∼ tissue*  *+*  *cell type*”. We additionally tested the effect of immune status of cell types on transcriptome conservation levels using a mixed model ANOVA via the *lme* function of the *R nlme* package (v.3.1-144) with transcriptome conservation set as the response variable, immune status as the explanatory variable and tissue as a random effect.

### Differential Gene Expression Analysis

For each cell type in each tissue, genes that showed ageing-related differential expression were identified using Spearman correlation between the expression level of the gene versus individual age (as implemented on bulk RNA-seq datasets). Due to higher noise in the scRNA-seq dataset compared to bulk RNA-seq datasets, a cut-off of *rho* (ρ) > 0.5 was used to define age-related genes, instead of using both *rho* and *P*-value cut-offs. Age-related genes with *rho* < −0.5 were classified as young-biased genes and those with *rho* > 0.5 were classified as old-biased genes.

### Transcriptome Conservation

Expression-conservation correlations and related analyses were conducted identically to those conducted on bulk RNA-seq data.

### Conservation Difference Between Old and Young-Biased Genes

To test whether the distribution of relative conservation scores differs between old-biased genes and young-biased genes, we calculated the MRCS of these gene sets identified per cell type in each tissue (i.e. one estimate per gene set per cell type). Then, across all cell types in each tissue, we applied the Wilcoxon signed rank test on the distributions of MRCS of old-biased and young-biased genes using the *wilcox.test* function with the parameter “*paired*  *=*  *TRUE“* in the *R stats* package.

### Immune Contributions to ADICT

To test whether immune cells behave differently in terms of age-related changes in sequence conservation than nonimmune cells, we calculated the difference between mean relative conservation scores of old-biased and young-biased genes for each cell type in the dataset by subtracting the mean relative conservation scores of old-biased genes from the mean relative conservation scores of young-biased genes. The list of cells used and their immune status is shown in Table S4. Next, we tested whether the distributions of MRCS differences and ADICT signals (Spearman correlation between expression-conservation correlation vs. age) differed between immune and nonimmune cells. For cell types that are present in multiple tissues, we used the mean of MRCS differences calculated in each tissue as the MRCS difference score for the cell type. We similarly used the mean *rho* scores across different tissues for repeating cell types when comparing ADICT signals. For statistical comparison, we used the Mann–Whitney *U* test as implemented by the *wilcox.test* function with default parameters in the *R stats* package. Additionally, we calculated effect sizes for the differences using Cohen's d, for both the MRCS differences and differences in ADICT signals (Spearman correlation distributions).

To study the contribution of immune genes to observed ADICT signals, we classified our gene set of genes as immune-related or nonimmune based on whether genes were associated with the GO term “immune system process” (GO:0002376) and compared the mean conservation scores of old- and young-biased immune-related genes with their respective nonimmune-gene counterparts for all cell types using Mann–Whitney *U* test. To test the overrepresentation of immune-related genes among the genes that contribute to ADICT, we calculated odds ratios for prevalence of immune-related genes in old-biased and low-conserved genes versus remaining old-biased genes; and young-biased and high-conserved genes versus remaining young-biased genes. For the statistical analysis, we used *fisher.test* function with default parameters in the *R stats* package and adjusted the *P*-values for multiple testing using Benjamini-Hochberg correction. Genes were classified into low- and high-conserved sets by taking the bottom and top quarters of genes in terms of conservation scores.

## Supplementary Material

evaf187_Supplementary_Data

## Data Availability

Only publicly available datasets were used in this study. All relevant accession numbers are provided in Table 1 and throughout the paper.

## Code Availability

The computer codes used in the study are available on https://github.com/NisanYildiz/selection-shadow/.
