# Supplementary material for: Under the Shadow: Old-biased Genes Are Subject to Weak Purifying Selection at Both the Tissue- and Cell Type-Specific Levels
Source: Genome Biol Evol. 2025 Oct 15;17(10):evaf187. doi: 10.1093/gbe/evaf187 (PMC12569596; doi:10.1093/gbe/evaf187)
Supplement: evaf187_Supplementary_Data [file evaf187_supplementary_data.zip › Yildiz-et-al-Supp-Figures-revised.pdf]

Supplementary Figures

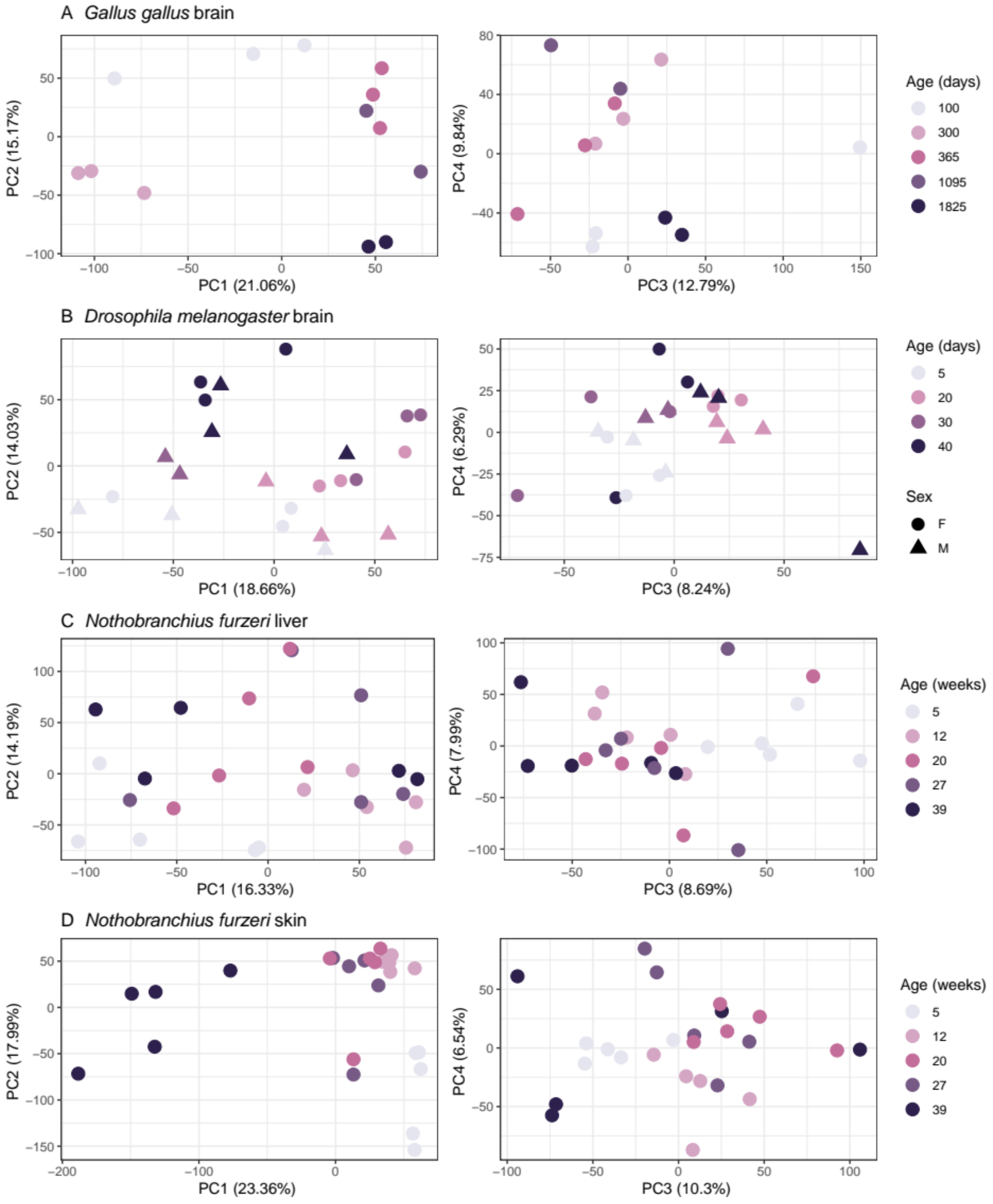

Figure S1. Principal component analysis (PCA) using expression levels of each dataset (*n* = [22405, 12344, 23064, 23178] genes for *G. gallus*, *D. melanogaster*, *N. furzeri* liver and *N. furzeri* skin, respectively). Only the first four PCs are plotted. Numbers in the parentheses show the percentage of variation explained by each PC. Age and sex (exist only for *D. melanogaster*) labels are indicated on the right of the plots.

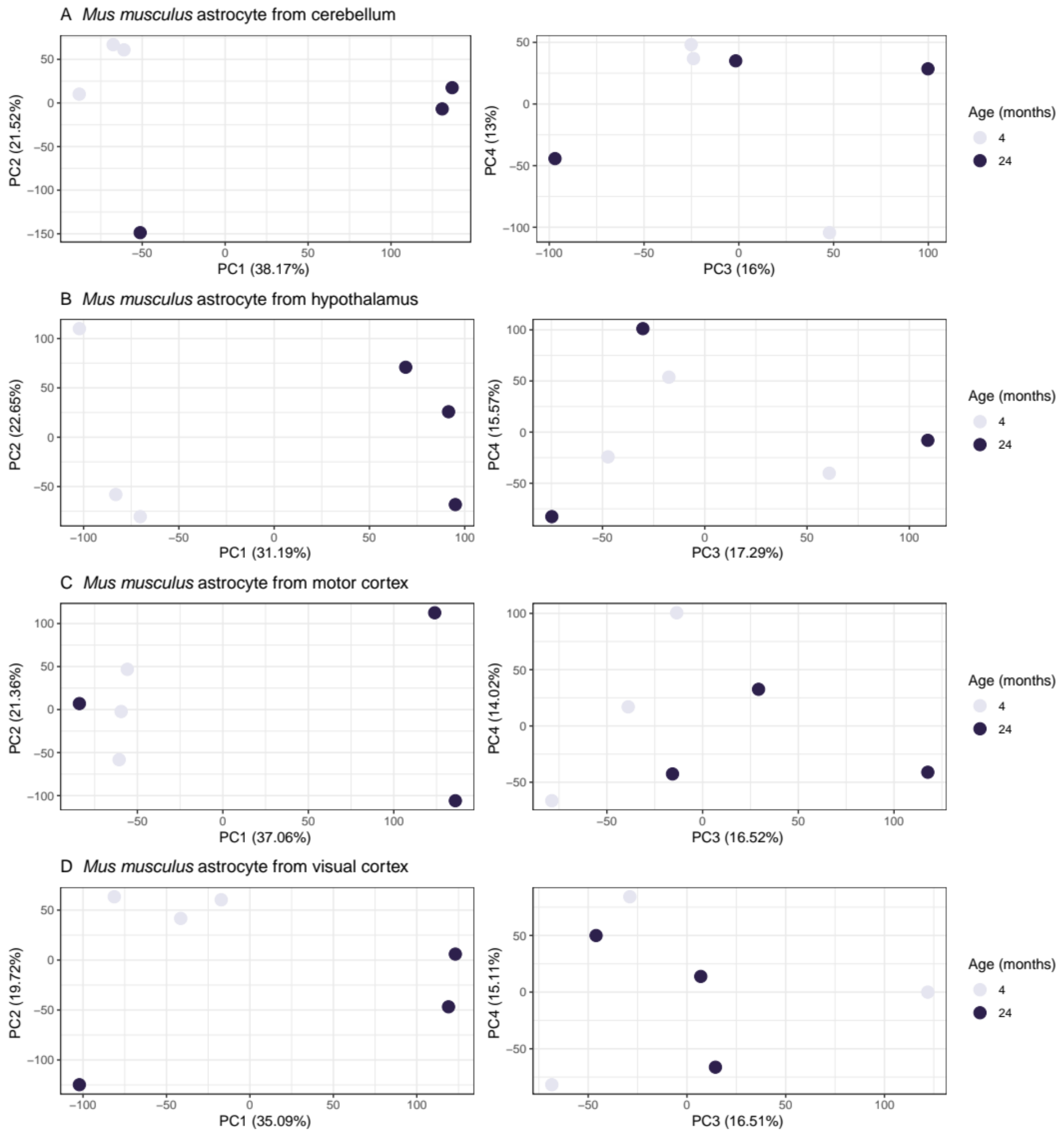

Figure S2. Principal component analysis (PCA) using expression levels of astrocyte-enriched *M. musculus* brain region samples from GSE99791 dataset (*n* = [28572, 28516, 27649, 27580] genes for cerebellum, hypothalamus, motor cortex and visual cortex, respectively). Only the first four PCs are plotted. Numbers in the parentheses show the percentage of variation explained by each PC. Age labels are indicated on the right of the plots.

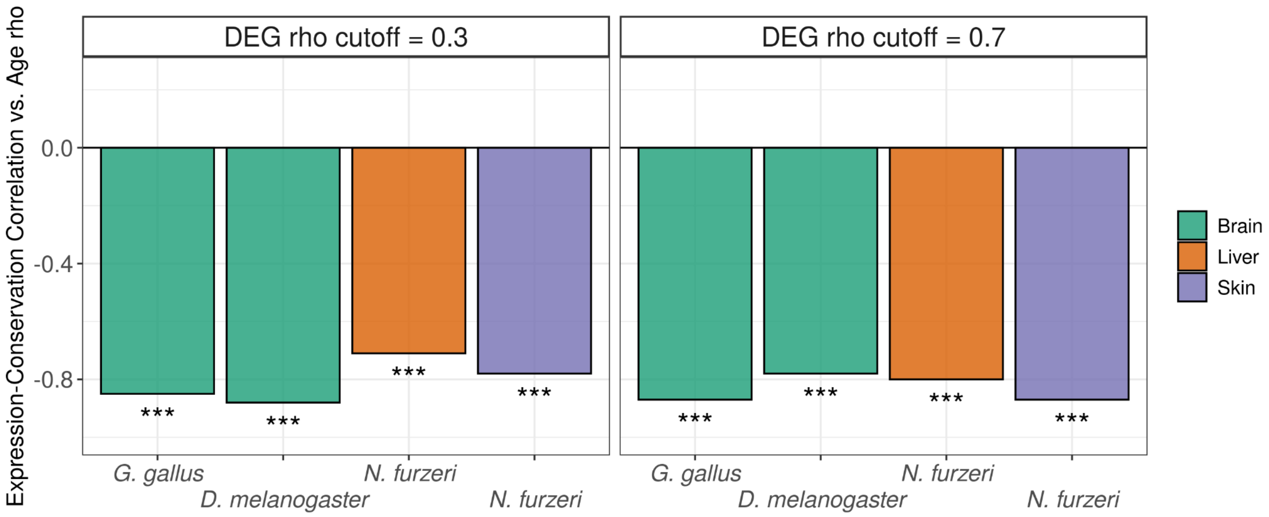

Figure S3. Summary of Spearman correlations between expression-conservation and age for different datasets using only differentially expressed genes (DEG), with two different (|rho| > 0.3 on the left and |rho| > 0.7 on the right) expression-age correlation cut-offs to be classified as DEG. (***): $p < 0.001$.

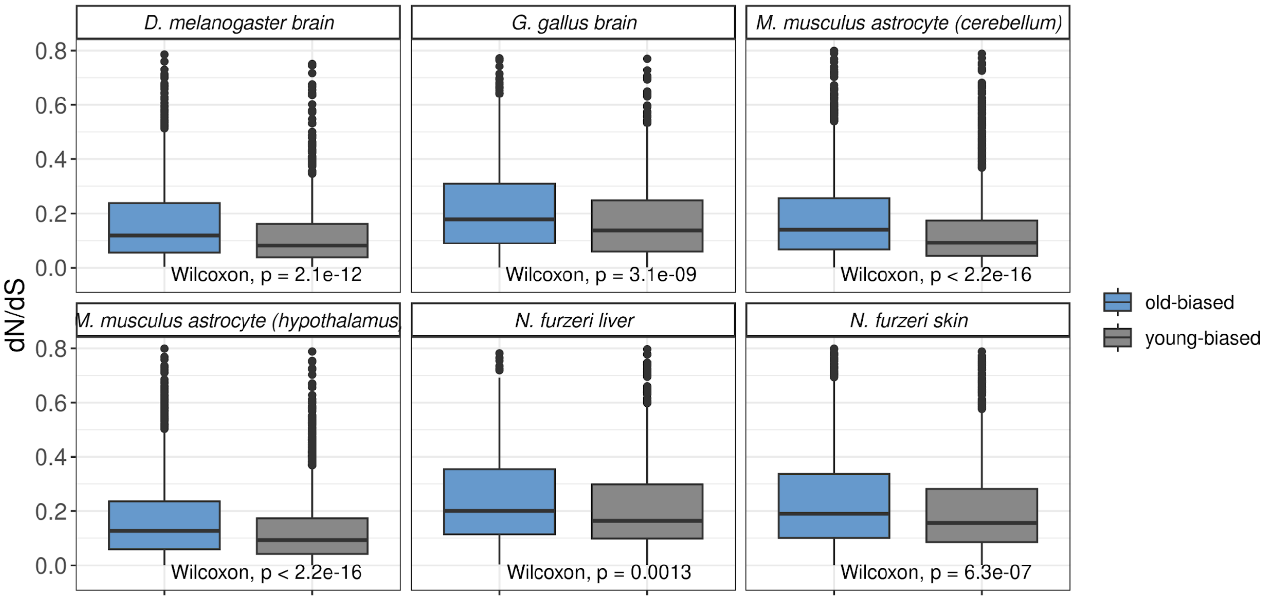

Figure S4. dN/dS values for old- vs. young-biased genes for bulk-tissue transcriptomes of *D. melanogaster*, *G. gallus, N. furzeri,* and astrocyte-enriched tissue transcriptomes of *M. musculus*. Insets represent Mann-Whitney U test p-values between old- and young-biased gene sets.

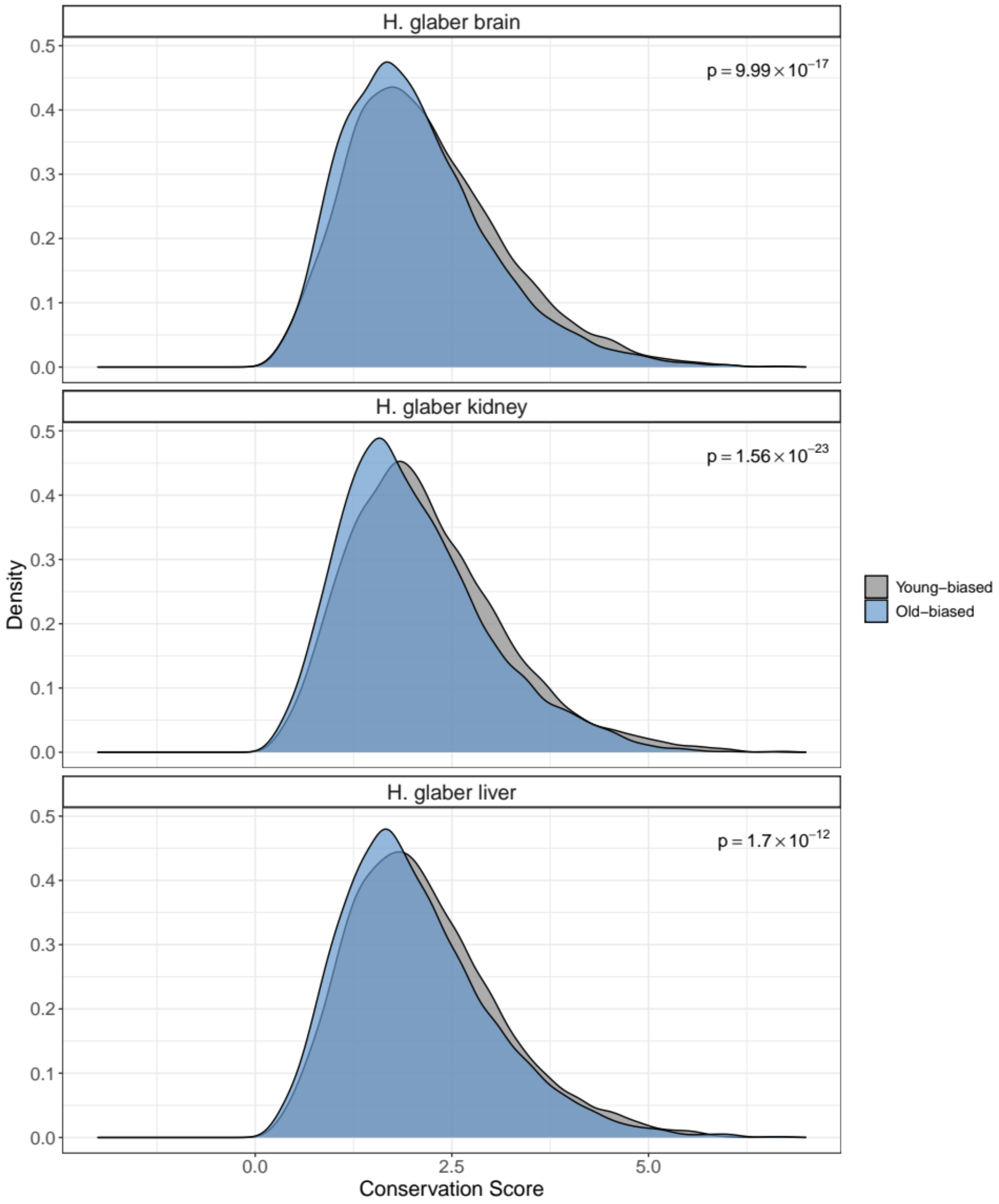

Figure S5. Distribution of the conservation scores for young-biased (gray) and old-biased (blue) genes in brain, kidney and liver of *H. glaber*. Old-biased genes have consistently lower conservation scores compared to young-biased genes across tissues. P-values indicate Welch's *t*-test results between young- and old-biased genes.

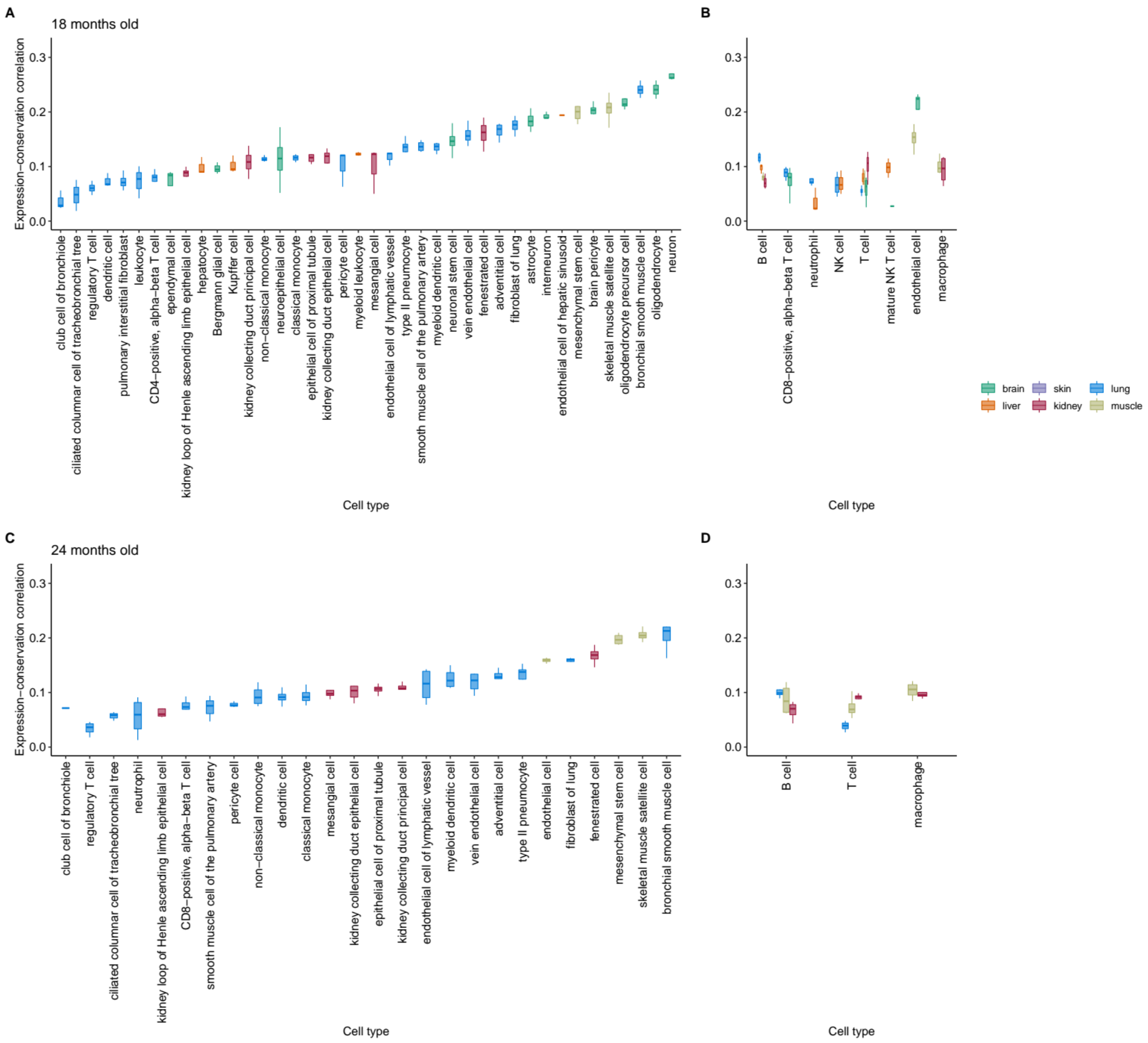

Figure S6. Variability of expression-conservation correlation levels across cell-type transcriptomes of 18-month-old (**A**, **B**) and 24-months-old (**C**, **D**) individuals. Each data point within bar plots represents Spearman's correlation values for an individual, calculated using gene expression values averaged across cells belonging to that individual. Panels A, C and B, D show cell-types only sampled in one tissue and cell-types sampled from multiple tissues, respectively. Color-coding indicates the tissues which the cell-types were sampled from (see key at the top of the figure). Cell types with less than three correlation values (i.e. individuals) are excluded.

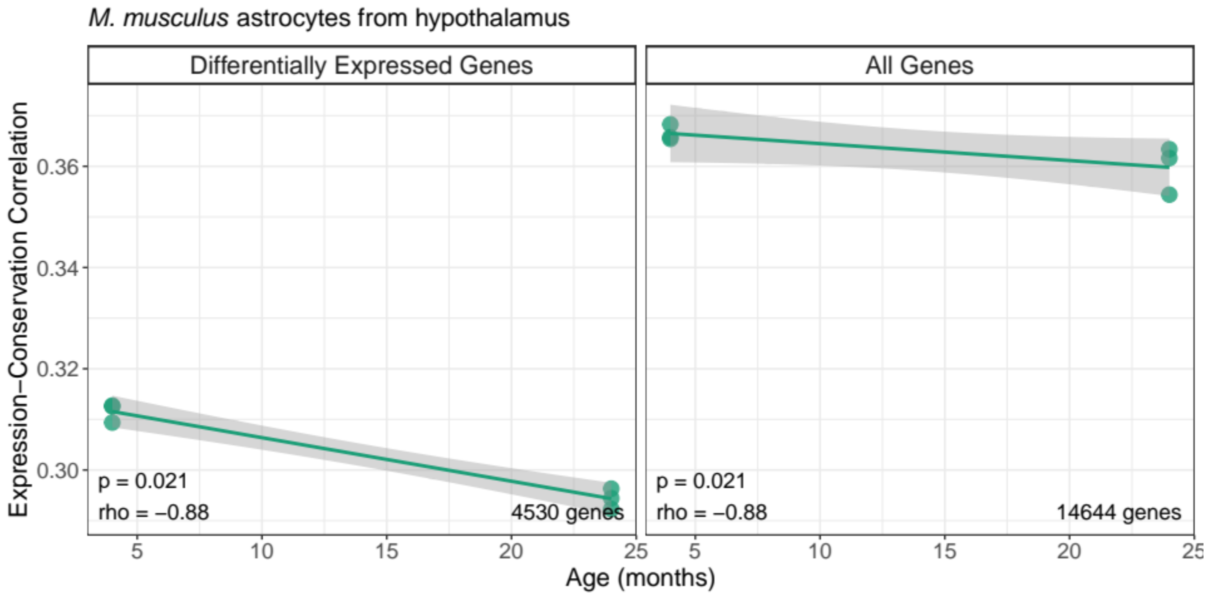

Figure S7. Age-related changes in expression-conservation correlation in *M. musculus* astrocyte transcriptome from the hypothalamus. The y-axis shows the Spearman correlation coefficient between expression level and protein sequence conservation metric across genes for each individual in this dataset (*n* = 6). The x-axis shows individual age. The left panel shows the analysis results using genes differentially expressed with age (*n* = 4,530), and the right panel shows the analysis results using the whole transcriptome (*n* = 14,644). *rho* and p-values in the inset indicate the results of the Spearman correlation between the expression-conservation correlation and age.

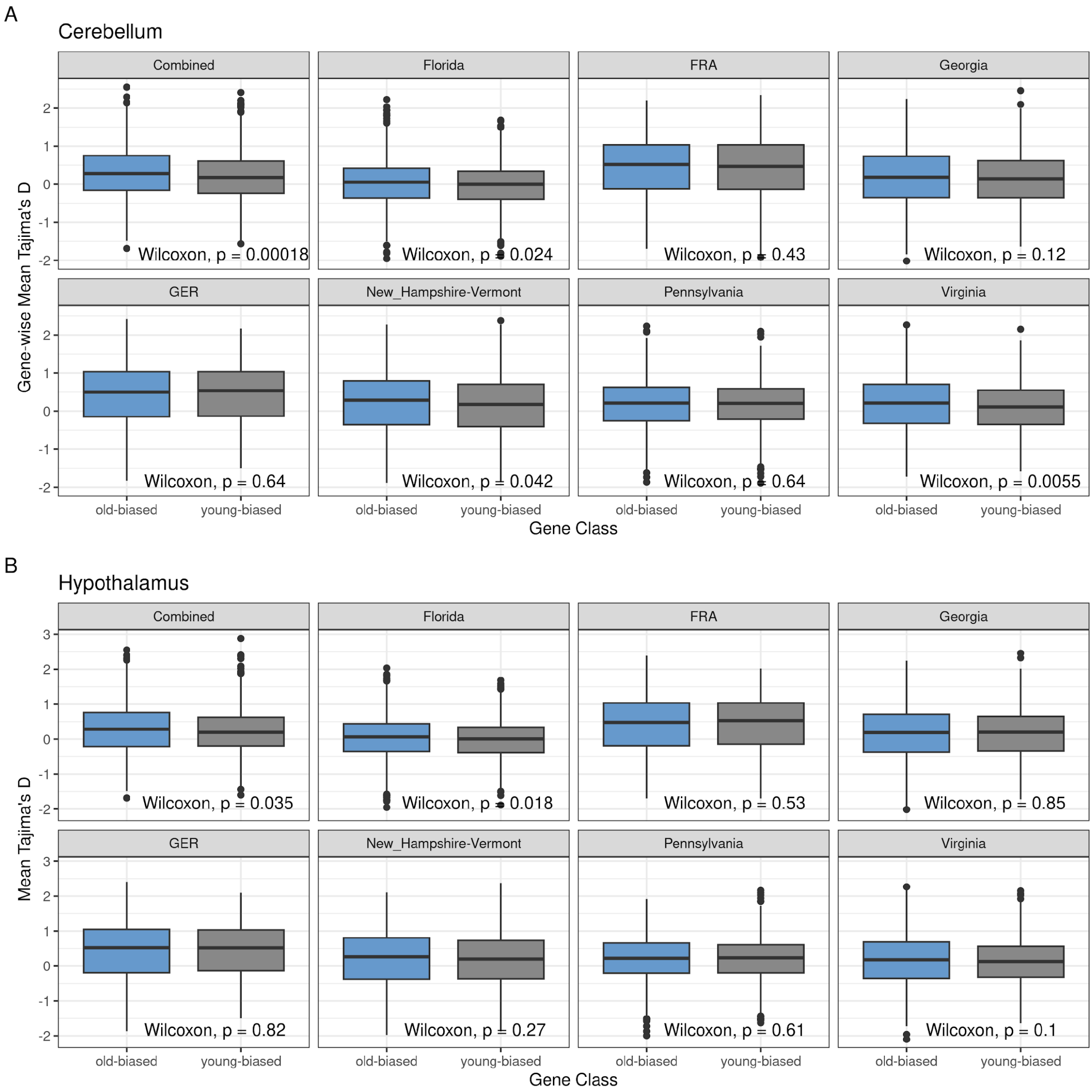

Figure S8. Tajima's D values of old- vs. young-biased genes, as classified on astrocyte-enriched (A) cerebellum and (B) hypothalamus transcriptomes. Tajima's D values are given for a "combined" group, treating the samples as a part of a single super-population; and for separate populations ("Florida", "France", "Georgia", "Germany", "New Hampshire / Vermont", "Pennsylvania", and "Virginia"). Insets represent Mann-Whitney U test p-values between old- and young-biased gene sets.
